# Co-registration of optoacoustic tomography and magnetic resonance imaging data from murine tumour models

**DOI:** 10.1016/j.pacs.2019.100147

**Published:** 2020-01-16

**Authors:** Marcel Gehrung, Michal Tomaszewski, Dominick McIntyre, Jonathan Disselhorst, Sarah Bohndiek

**Affiliations:** a Department of Physics, University of Cambridge, UK; b Cancer Research UK Cambridge Institute, University of Cambridge, UK; c Werner Siemens Imaging Center, Preclinical Imaging and Radiopharmacy, University of Tuebingen, Germany

**Keywords:** Optoacoustic tomography, Magnetic resonance imaging, Image registration

## Abstract

As optoacoustic tomography (OT) emerges as a mainstream pre-clinical imaging modality, understanding the relationship between optoacoustic and other imaging biomarkers in the context of the underlying tissue biology becomes vitally important. Complementary insight into tumour vasculature and hypoxia can be gained using OT alongside magnetic resonance imaging (MRI)-based techniques. To evaluate the relationship between these metrics and the relative performance of the two modalities in assessment of tumour physiology, co-registration of their output imaging data is required. Unfortunately, this poses a significant challenge due to differences in animal positioning during imaging. Here, we present an integrated framework for registration of OT and MR image data in mice. Our framework combines a novel MR animal holder, to improve animal positioning during imaging, and a landmark-based software co-registration algorithm. We demonstrate that our protocol significantly improves registration of both body and tumour contours between these modalities, enabling more precise multi-modal tumour characterisation.

## 1 Introduction

Optoacoustic tomography (OT) is an emerging imaging modality, able to reveal the distribution of tissue optical absorption coefficient in real-time with a spatial resolution of ∼180 μm at ∼3 cm penetration depth [1]. Thanks to the distinct optical absorption profiles of oxy- and deoxyhaemoglobin, acquiring OT data at multiple wavelengths (multispectral optoacoustic tomography, MSOT) makes it possible to derive optoacoustic imaging biomarkers of total haemoglobin concentration (THb) and blood oxygenation (SO_2_) [2]. Application of these functional MSOT imaging biomarkers has been shown to provide insight into both the architecture and function of the blood vasculature, for example, in cancer imaging, where it can be used to monitor tumour development [3], [4] and detect response to therapy [5], [6].

Functional imaging of the blood vasculature is also possible with a wide range of magnetic resonance imaging (MRI)-based techniques. Taking the example of cancer imaging, dynamic contrast enhanced (DCE) MRI [7], blood oxygen level dependent (BOLD) [8], oxygen enhanced (OE) MRI [9] and arterial spin labelling (ASL) MRI [10] have all been demonstrated to provide insight into tumour blood vessel function and the surrounding tissue hypoxia.

MSOT-measured THb and SO_2_ biomarkers have been shown to indicate tumour vascular maturity [4], while functional MR methods provide complementary measures of vascular perfusion and permeability [7]. Both approaches can also provide insight into tissue hypoxia. While MSOT lacks the outstanding anatomical soft tissue information and versatility of the multiple MRI contrast mechanisms, it does provide whole body functional imaging in small animals at high spatio-temporal resolution with a diverse range of opportunities for molecular imaging using contrast agents [11]. Combining the strengths of these complementary imaging modalities can therefore provide detailed insight into the functional characteristics of tumour vascular phenotypes as well as tissue hypoxia. Further, important translational questions arise as to whether and if so how these complementary imaging biomarkers relate and which provide the best sensitivity for *in vivo* cancer imaging.

The correct combination of spatial information from different imaging modalities requires careful alignment of the images and hence an efficient co-registration algorithm. This is usually achieved in both patient and small-animal imaging by careful body positioning and scanning process optimisation, aided by software-based alignment. Well-established, clinically used solutions are available [12], [13] and provide excellent results for fusion of positron emission tomography (PET), computed tomography (CT) and MRI data. Unfortunately, modalities such as OT that involve different positioning of the animal or patient pose a significant challenge to co-register. As MSOT typically requires suspending the animal under water, the distribution of external forces exerted on the animal are distinct from forces acting in conventional imaging, leading to deformations that need to be corrected. Successful co-registration of OT and MR images has been reported previously in the brain of small animals [14], [15], however, being contained within the skull, the brain is not subject to any deformation due to external forces, making it a relatively simple organ to co-register.

Here, we present a new integrated framework for registration of MSOT and MR image data in pre-clinical studies of mice, which can be applied to soft, deformable tissues such as tumours. The method combines a novel animal holder design and a robust co-registration algorithm. We first describe the method and show its performance for co-localization of the internal tumour structures between the modalities. We then demonstrate the improvement in co-registration achieved by the combination of hardware and software-based solutions, compared to the manual overlay of the tumour regions with standard animal holders used for MSOT and MRI. Finally, we demonstrate the application of the co-registration framework for comparison of perfusion-based data recorded using MSOT and MRI.

## 2 Methods

### 2.1 Animal experiments

All animal procedures were conducted in accordance with project (70-8214) and personal license (IDCC385D3) issued under the United Kingdom Animals (Scientific Procedures) Act, 1986 and were approved locally under compliance form number CFSB0671. Subcutaneous tumours were established in male BALB/c nude mice (Charles River, 7–10 weeks old, 17–22 g) by inoculation of cells from one of three different cancer cell lines in both flanks (1.5 × 10^6^ LNCaP prostate adenocarcinoma cells, n = 3 mice; 1.5 × 10^6^ PC3 prostate adenocarcinoma cells, n = 3 mice; 1 × 10^6^ mouse K8484 pancreatic adenocarcinoma cells, n = 3 mice) in 100 *μ*L phosphate buffered saline (PBS). Using three different cell lines allowed us to investigate the co-registration procedure across a range of morphological and functional characteristics.

### 2.2 Multispectral optoacoustic tomography (MSOT)

An MSOT inVision 256-TF commercial small animal imaging system (iThera Medical GmbH) was used. Briefly, a tunable optical parametric oscillator (OPO) pumped by an Nd:YAG laser provides excitation pulses with a duration of 9 ns at wavelengths from 660 nm to 1200 nm at a repetition rate of 10 Hz with a wavelength tuning time of 10 ms and a peak pulse energy of 90 mJ at 720 nm. Ten arms of a fibre bundle provide uniform illumination of a ring-shaped light strip of approximately 8 mm width. For ultrasound detection, 256 toroidally focused ultrasound transducers with a centre frequency of 5 MHz (60% bandwidth) are organized in a concave array of 270 degree angular coverage and a radius of curvature of 4 cm.

Mice were prepared according to our standard operating procedure [16]. Each mouse was anaesthetised using<3% isoflurane and moved into a custom animal holder (iThera Medical GmbH), wrapped in a thin polyethylene membrane, with ultrasound gel (Aquasonic Clear, Parker Labs) used to couple the skin to the membrane. The holder was then placed within the MSOT system and immersed in degassed water maintained at 36 °C. The mouse was allowed to stabilise for 15 min within the system prior to initialisation of the scan and its respiratory rate was then maintained in the range 70–80 bpm with ∼1.8% isoflurane concentration for the entire scan. The imaging slice was chosen to show largest cross-sectional area of the tumours on one or both flanks where possible. The position of the slice relative to the tumor edges was noted. Images were acquired in the single slice using 10 wavelengths between 700 nm and 880 nm and averaging of signals from 6 pulses per wavelength; a single slice acquisition was 5.5 s in duration. For Oxygen Enhanced Optoacoustic Tomography, 70 such images were acquired continuously, with the breathing gas switched from medical air (21% Oxygen) to pure oxygen (100% Oxygen) after 30 scans, for the purpose of quantification of the response in blood oxygen saturation to such defined oxygen challenge.

### 2.3 Magnetic resonance imaging (MRI)

A 9.4 T Agilent MRI system (Agilent, Santa Clara, USA) running VnmrJ 3.1, using an Agilent quadrature transmit/receive millipede volume coil of 38 mm inner diameter was used. The same anaesthesia protocol as for optoacoustic imaging experiments was maintained. A physiological monitoring system was used for observing mouse status and for sequence triggering (SAII, Stony Brook, NY, USA). The core temperature of the mouse was monitored using a rectal probe, and stabilized to 37 °C using an air heating system. Firstly, coronal multislice T2-weighted images were acquired covering the entire tumour using a respiratory-gated fast spin-echo sequence (field of view 40 mm, slice thickness/gap 0.95/0.05 mm, 256×256 points, TR 2000 ms, echo spacing 9 ms, echo train length 8, effective TE 36 ms, 2 averages, 3–4 slices acquired per gate) with chemical-shift-selective fat suppression. Based on these maps, axial imaging slices were chosen, such that centre slice would best match the MSOT imaging slice based on the slice with highest cross-sectional area and distances from tumor edges matching these observed in MSOT. The axial T2 weighted images were then acquired covering the entire tumour, using a fast echo sequence with parameters as above.

Dynamic contrast enhanced (DCE)-­MRI data were acquired using a spoiled gradient echo sequence (field of view 40 mm, 2 mm slice, 128×128 points, TR 20 ms, TE 1.62 ms, 2 averages). 10 images were acquired during the 1 minute prior to administration of contrast agent (Gadavist, Bayer, 200 μmol/kg) to provide a baseline reference and 120 images were acquired in the 11 min after injection.

### 2.4 Hardware for improved co-localization

**Fig. 1:**
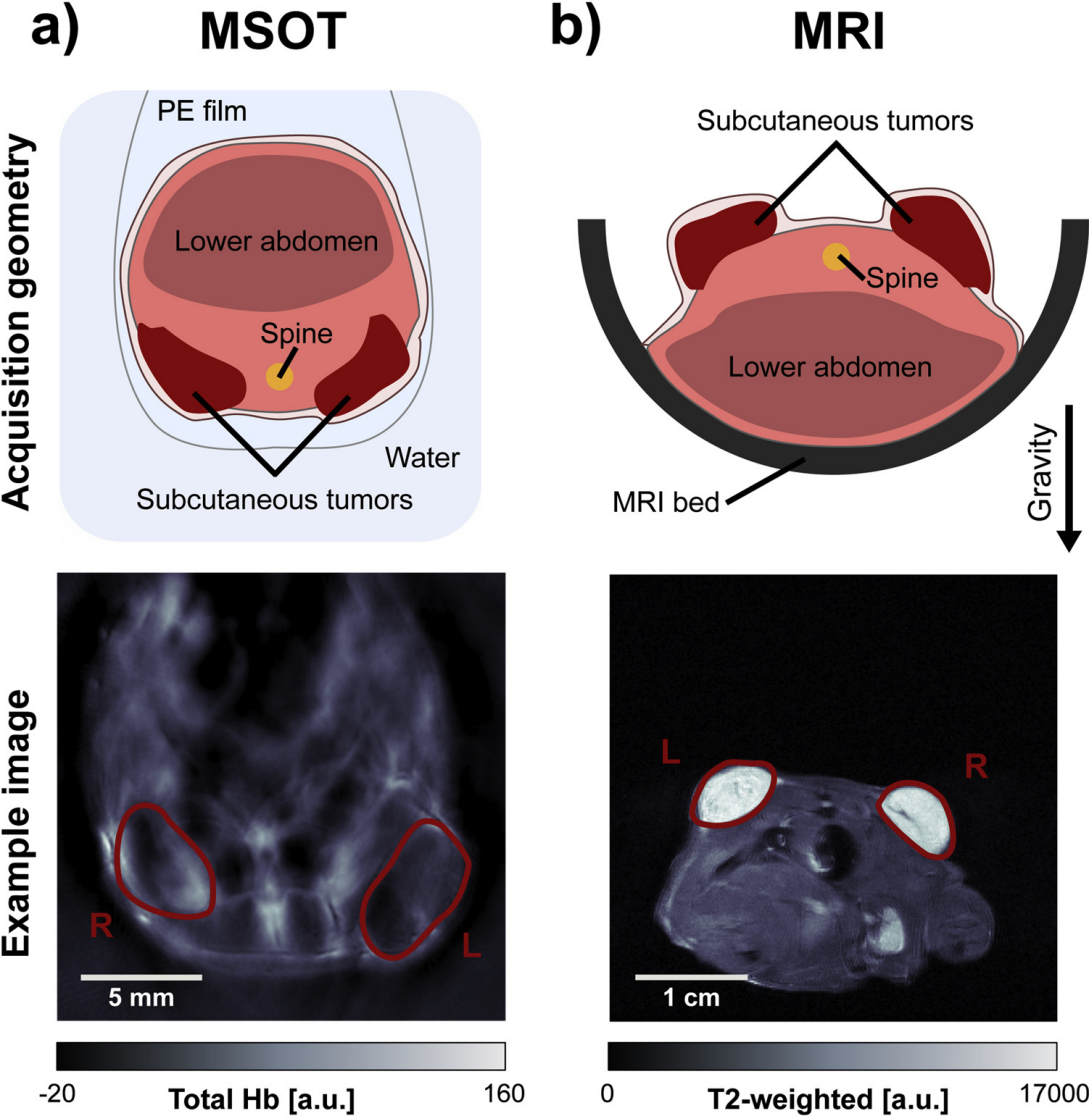
Conventional MSOT and MRI holder geometries. (a) Animal holder geometry and example image showing the total haemoglobin signal after spectral unmixing, acquired using MSOT. (b) Conventional animal holder geometry and example fast spin-echo image from MRI.

**Fig. 2:**
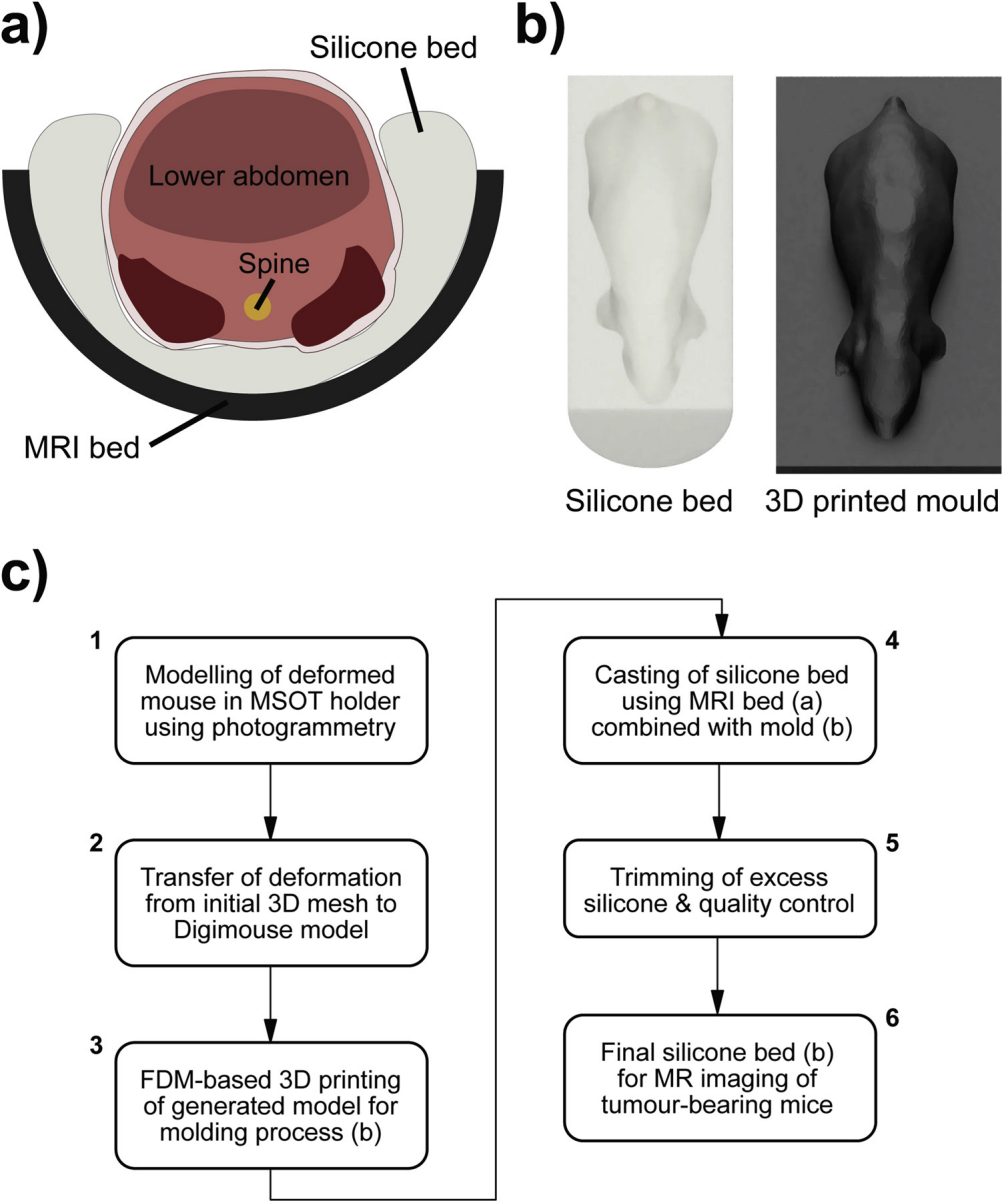
Novel MRI holder geometry. (a) Design of the silicone holder to achieve a comparable anatomical cross-section within MRI and MSOT. (b) Rendering of the silicone bed and the corresponding 3D printed mold. (c) Flowchart with individual steps for generation of the silicone bed (FDM = Fused Deposition Modeling).

To facilitate co-registration of MSOT and MRI data, a new mouse holder was developed to reproduce the spatial positioning and body deformation of the MSOT (Fig. 1a) during the MRI acquisition as accurately as possible. This was achieved using a silicone bed (Fig. 2a) for MRI imaging. The main objective of the design process was to closely resemble the anatomical positioning in the MSOT device. The holder was fabricated based on photogrammetry, a process which allowed for a 3D model of the mouse suspended in PE film to be produced based on a set of pictures taken from different angles. A point cloud is generated from these images by aligning the camera positions with subsequent conversion into a mesh, performed with the software 3DF Zephyr v3.5 (3DFLOW, Italy). Due to the low resolution of the mesh, the estimated deformation was transferred to a 3D model (Fig. 2b) from the Digimouse atlas [17] using the software Fusion 360 (Autodesk, US). The resulting model was converted into a mold (by a adding a baseplate) in STL file format, which is available on a GitHub repository together with an additional model to accommodate different tumour sizes. The result was then printed with Polylactic acid (PLA) using an Anet A6 3D printer (Anet, China), instructed with the slicer software Ultimaker Cura 2.6 (Ultimaker, Netherlands). The 3D printed mold was inserted into a conventional MRI bed, with two circular disks as silicone leakage blocker, and the resulting cavity was filled with an equal mixture of A and B component of the chosen silicone (Polycraft T15 Translucent Silicone, MB Fibreglass) before being cured for 24 h. Extra soft silicone (Shore Hardness A15) was used to increase compressibility in order to accommodate subtle alterations in individual mouse anatomies. Subsequently, the negative mold was removed, the silicone bed taken out and the excess silicone trimmed with a blade. The full process is summarised in a flow chart in Fig. 2c.

After imaging in the MSOT, mice were maintained under anaesthesia and transferred for MRI. A subset of n=4 mice (3 LNCaP tumour bearing and 1 PC3 tumour bearing) underwent MRI placed in the prone position in a half-pipe plastic holder, with the tumours on the back facing upwards using the conventional MRI holder geometry (Fig. 1 b).

The remaining 5 animals (2 PC3 tumour bearing and 3 K8484 tumour bearing) were scanned in the custom silicone holder (Fig. 2). Transfer into the silicone MRI bed was made in a smooth motion while maintaining the supine orientation of the mouse to preserve the positioning. The silicone bed showed a large, broad nuclear magnetic resonance excitation at 7 ppm upfield of water, which was clearly visible in fast spin-echo images. Image registration was greatly simplified by suppressing this signal, as the silicone is not present in the MSOT image data. Therefore, for imaging sessions employing the silicone bed, a modified chemical-shift selective fat suppression sequence was employed, using a sinc pulse of bandwidth 3 kHz centred at 2 kHz from the water peak, between the fat and silicone resonances, to suppress both fat and silicone.

### 2.5 Software co-registration

The main objective of any general co-registration software is to merge the coordinate system of the moving image *I_M_* with the fixed (or reference) image *I_F_*. The transformation matrix T is used to warp the moving image in order to minimise the error metric with the fixed image. This process is iterated until a certain convergence criterion is reached.

A landmark-based co-registration approach [18] based on non-reflective similarity with the addition of optional reflection was used to register the tumour areas between modalities, utilising a set of prominent anatomical features including the tumour edges and spine location as landmarks. The positions of these features, identified manually, were denoted in the MR image as *I_M_* and the matching positions in the MSOT image as *I_F_*. These vectors were then used to calculate the transformation matrix and transform both modalities into the same coordinate space, minimising the euclidean distance between the landmarks. A landmark-based approach was chosen to maximise robustness. High structural homogeneity of xenograft tumours and their regular shape can limit the performance of other, internal information or shape based [19] algorithms.

**Fig. 3:**
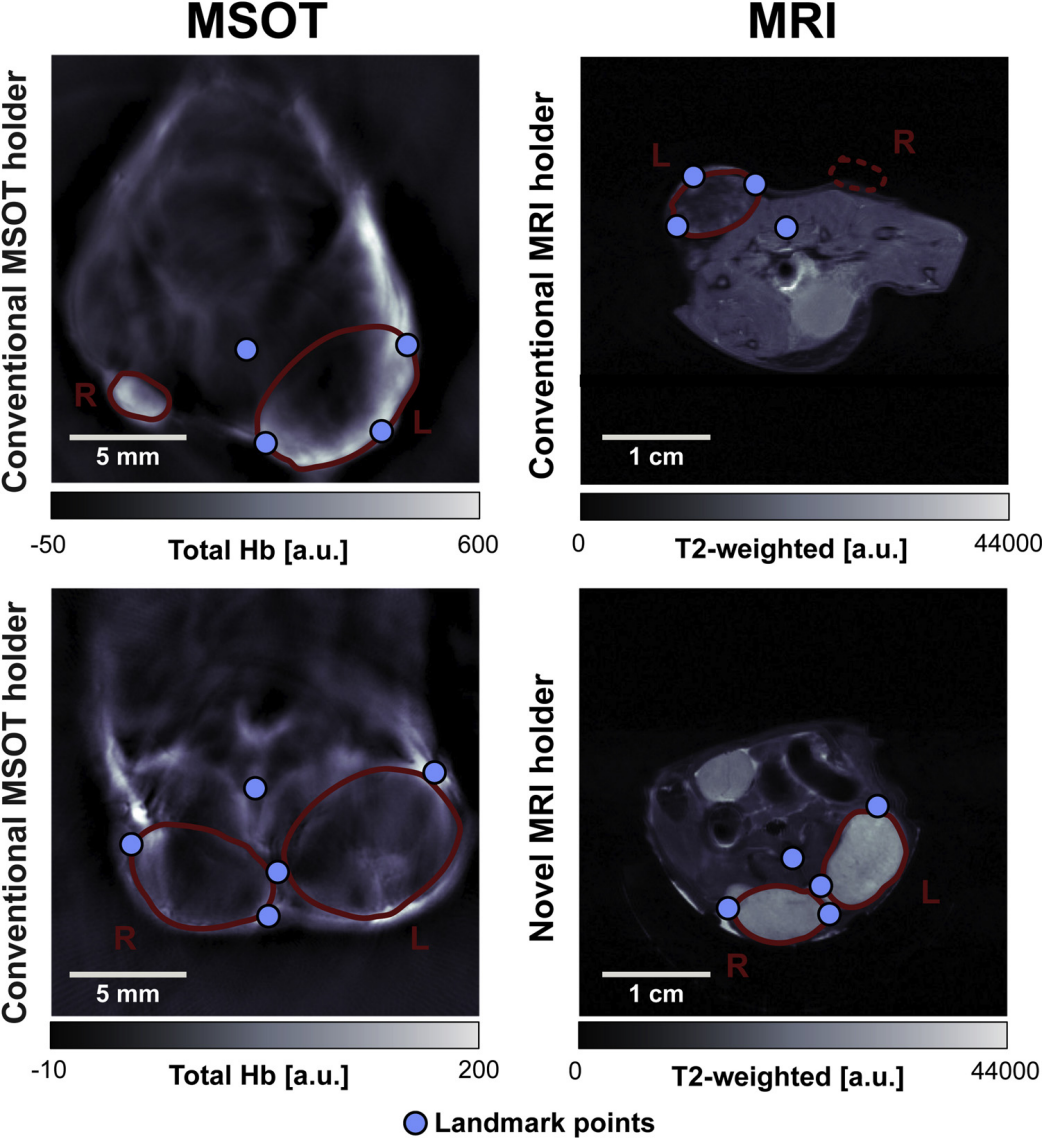
Qualitative comparison of tomographic image data from OT and MRI modalities using the conventional and novel MRI holders. *Top*: MSOT/MRI image pair with conventional holder geometry as in Fig. 1. Tumour R is not visible in the plane of the corresponding MR image. *Bottom*: MSOT/MRI image pair with novel holder geometry as in Fig. 2.

The registration procedure was implemented in two steps, each constituting a landmark-based co-registration process. The first step ensured proper alignment between the body contours in MSOT and MRI, which provides constrained initial conditions to the tumour alignment, while the second step provided further alignment of the tumours. Landmarks for the first step were the spine and characteristic anatomical features visible in MRI and MSOT, such as contact points between tumours and body (Fig. 3). Second step landmarks were defined by points along the outline of the tumour: up to two points on an axis between the tumour and mouse body; and up to two points on the perpendicular axis (Fig. 5). The corresponding similarity-based transformation matrix was calculated using the MATLAB function *fitgeotrans*.

### 2.6 Image and statistical analysis

All image analysis was performed in MATLAB 2016a (Mathworks) using the Image Processing Toolbox, the Computer Vision Toolbox and custom scripts unless otherwise stated. All image data and custom analysis codes will be made openly available at doi: 10.17863/CAM.39741.

Image reconstruction was performed using an acoustic backprojection algorithm (iThera Medical GmbH) with an electrical impulse response correction, to account for the frequency dependent sensitivity profile of the transducers. Images were reconstructed with a pixel size of 100 μm × 100 μm which is approximately equal to half of the in-plane resolution of the InVision 256-TF. It should be noted that the out-of-plane resolution of this system is approximately 0.9 mm [20]. Pseudoinverse matrix inversion (*pinv* function in MATLAB 2016a) was applied to the measured optoacoustic spectrum in each pixel to calculate the relative oxy- [${\mathrm{HbO}}_{2}$] and deoxy-haemoglobin [*Hb*] signal. The presented images illustrate the total haemoglobin signal [${\mathrm{HbO}}_{2}+\mathrm{Hb}$] unless otherwise stated. Apparent blood oxygen saturation ${\mathrm{SO}}_{2}^{\mathrm{MSOT}}$ was calculated as a ratio of oxy- to total haemoglobin [4].

All MR images were flipped horizontally prior to image registration. The position of the slice analysed was chosen by the operator to best match the location of the imaging slice in OT, acquired directly before the MRI.

The analysis of registration accuracy of body and tumour contours was performed by calculating the Dice Similarity Coefficient (DSC). This coefficient is defined as:

$$\mathrm{DSC}=\frac{2|X\cap Y|}{\left|X|+|Y\right|}$$,

which allows quantification of the overlap between two binary masks, X and Y (i.e. original MRI body/tumour mask and the mask obtained from the MSOT image after co-registration). The higher the DSC, the better the overlap between the two binary masks and therefore, the more accurate the image registration result.

The results were compared on a per-tumour basis. Differences in DSCs between conventional and novel holder geometries as well as before and after landmark-based registrations (for body and tumour) were statistically tested with two-tailed paired t-tests (in the case of equal variances between sets of samples) and two-tailed unpaired t-tests (in the case of unequal variances between sets of samples). Data are reported as median ± standard deviation, unless otherwise stated.

DCE-MRI signal area under the curve (AuC) 1 min after contrast administration was compared to the change in blood oxygen saturation (${\mathrm{SO}}_{2}^{\mathrm{MSOT}}$) as measured by Oxygen Enhanced Optoacoustic Tomography in response to an oxygen challenge [4]. The tumour pixels were binarised into ‘responding’ and ‘non-responding’. The pixels were classified as responding when the difference between the average ${\mathrm{SO}}_{2}^{\mathrm{MSOT}}$ under air breathing and the last 20 frames (under oxygen breathing), exceeded twice the standard deviation of the ${\mathrm{SO}}_{2}^{\mathrm{MSOT}}$ under air breathing (the first 20 frames). The maps of such calculated response was co-registered with the DCE MRI AuC maps. The median DCE AuC values in the regions showing positive MSOT response and the rest of the tumour area were compared.

## 3 Results

### 3.1 Novel holder geometry improves visual anatomical similarity between MSOT and MRI

Visual inspection of MRI and MSOT images acquired with the conventional and novel MRI holder geometries yielded distinct differences in body shape and anatomical appearance (Fig. 3). Overall body shape and relative tumour location were not easily comparable for tumours imaged with the conventional protocol, while the new holder showed a high degree of similarity in body contour and tumour locations. A quantitative comparison of the contours of the mouse bodies in MSOT and MRI images using Dice similarity coefficients (DSCs) showed significant improvement (p=0.03, unpaired t-test) with the novel MRI holder, resulting in a higher DSC (0.63 ± 0.05 vs. 0.52 ± 0.07, novel vs. conventional). The higher DSC indicates that the novel MRI holder more accurately represents the body deformation observed in the MSOT.

### 3.2 Landmark-based contour registration improves animal body alignment

**Fig. 4:**
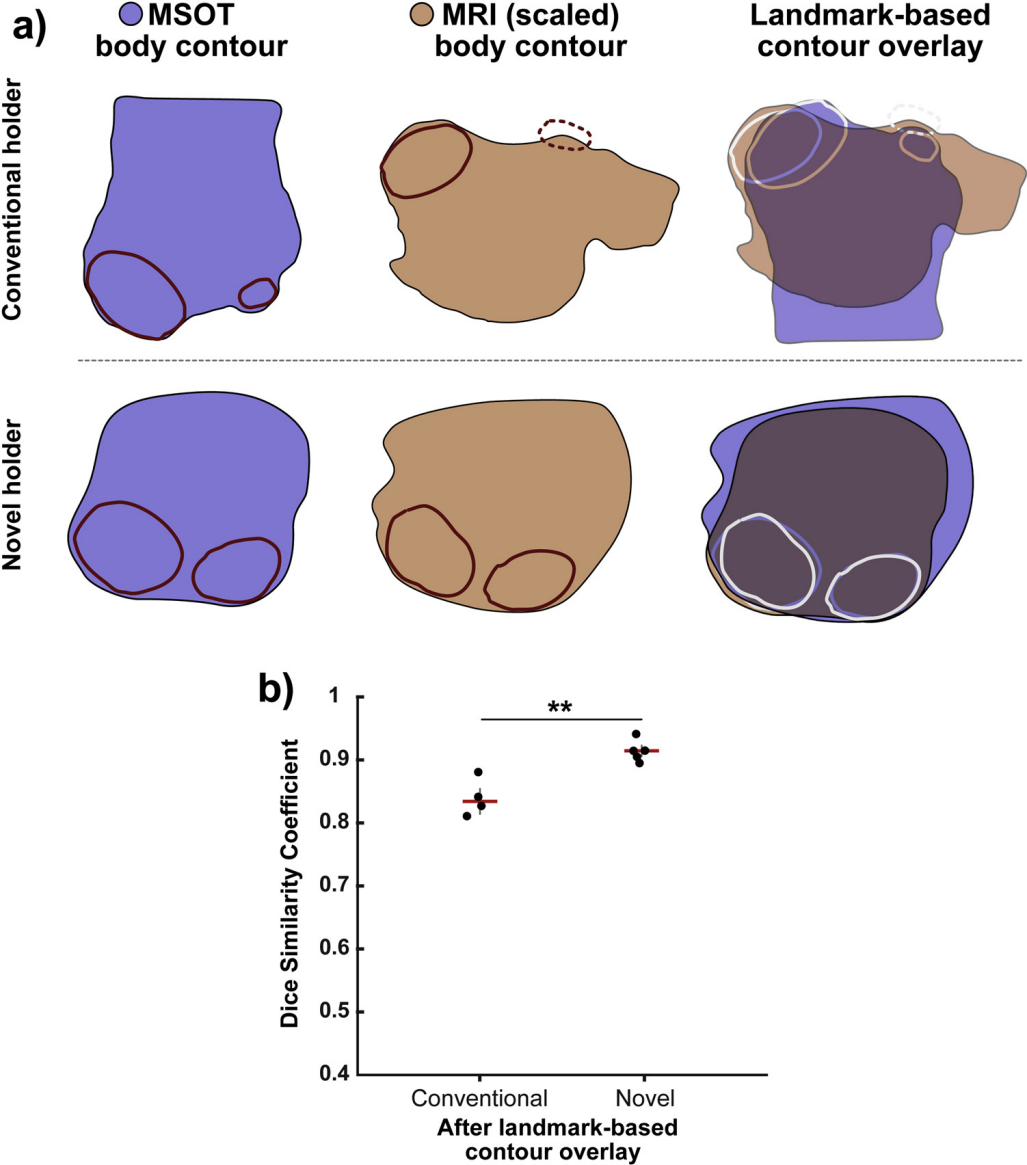
Comparison of body contour overlays of MRI/MSOT image pairs for conventional and novel holder geometries. (a) Body contour pairs (derived from binarized body outlines) for MSOT and MRI. Use of the novel protocol improves the agreement substantially (overlaid MSOT and MRI contours shown in blue and orange respectively). (b) Quantitative comparison of Dice similarity coefficient (n=4 for conventional holder and n=5 for novel MRI holder). * p<0.05, ** p<0.01, *** p<0.001 by unpaired two-tailed t-test (unequal variances) and paired two-tailed t-test (equal variances).

Landmark-based contour registration was then applied to MSOT and MR images acquired using both the conventional and novel MRI holders (illustrated in Fig. 4a). The difference between the conventional and novel MRI holders was more significant (p=0.002, unpaired t-test) following landmark-based registration with further improved DSCs (0.92 ± 0.02 vs. 0.83 ± 0.03, novel vs. conventional). In total (Fig. 4b), the contour registration procedure improved DSCs for body contour overlay significantly, both for the conventional (ΔDSC = 0.31, p=0.004, paired t-test) and novel (ΔDSC = 0.29, p = 7.2 $\times 10^{-5}$, paired t-test) holder. Comparing the performance of three operators (one expert, two non-experts) resulted in very robust results post transformation with DSC standard deviations ranging from 0.0057 to 0.0276, and an average standard deviation of 0.0157 by taking the square root of the averaged variances.

### 3.3 Landmark-based tumour contour optimisation further improves local anatomical similarity

**Fig. 5:**
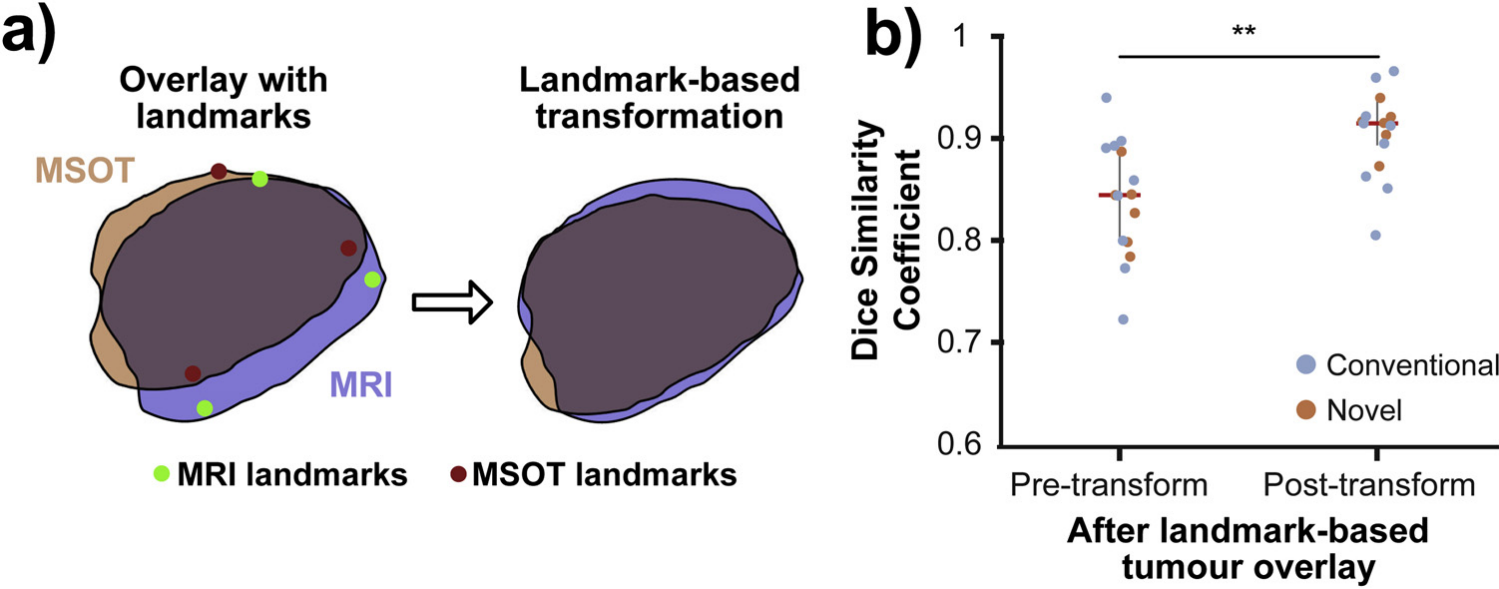
Overlays of tumour contours from MRI/MSOT image pairs before and after landmark-based optimization. (a) Comparison of a tumour outline before and after landmark-based tumour registration. (b) Quantification of the improvement in Dice similarity coefficient (n=15 tumours, combined data for conventional and novel MRI holder geometry). ** p<0.01 by paired two-tailed t-test (equal variances).

Following the body contour registration, each tumour was individually co-registered as an additional optimisation step. The tumour contours showed a qualitatively higher agreement after this additional landmark-based optimisation. The gain in registration accuracy was estimated to be between 5 and 15 pixels (375 μm–1125 μm), based on the distances between the co-registered tumour outlines (Fig. 5a). Quantitative assessment also showed in a significant improvement in tumour mask overlay DSCs (p=0.005, paired t-test) after landmark-based transformation of tumour masks (pre-transform: 0.85 ± 0.06 vs. post-transform: 0.92 ± 0.04, Fig. 5b). When comparing the performance of three operators (one expert, two non-experts), we obtained consistent results post transformation with strong robustness. This was quantified by DSC standard deviations ranging from 0.0079 to 0.0409, and an average standard deviation of 0.0257 by taking the square root of the averaged variances.

### 3.4 Application of the co-registration framework for comparison of data acquired using MSOT and MRI

**Fig. 6:**
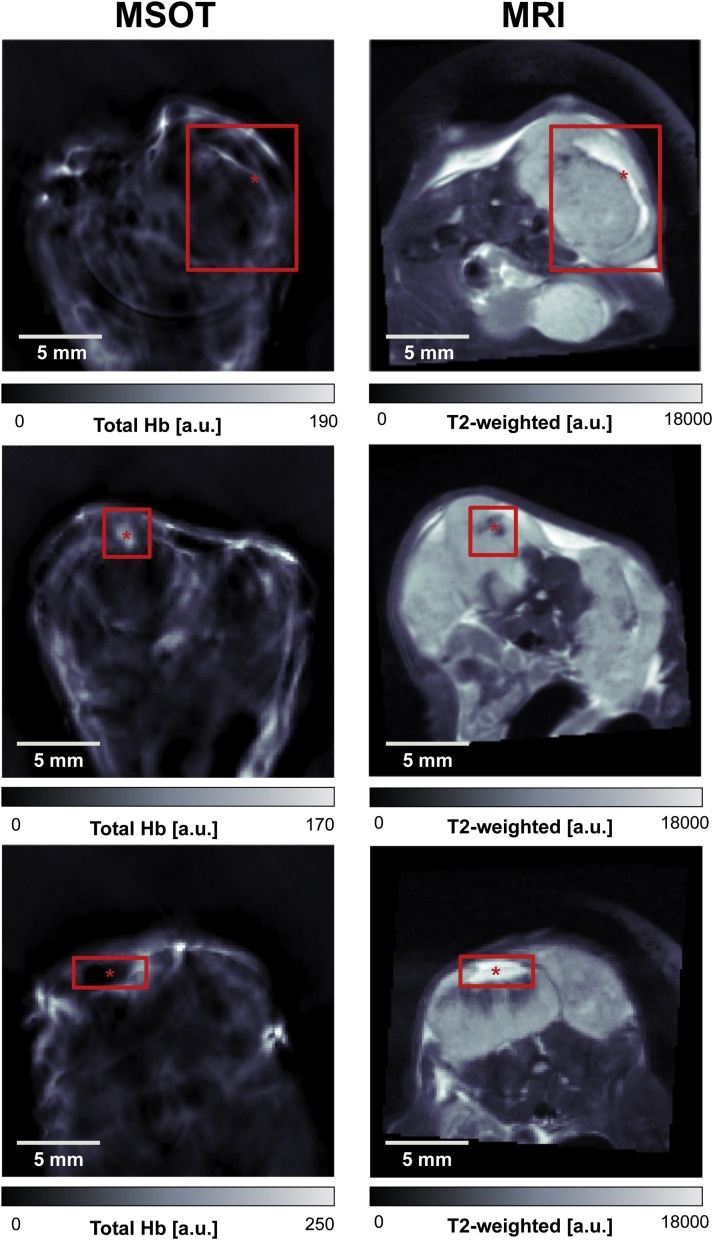
Anatomical features in three K8484 tumours after landmark-based body and tumour contour registration. The red asterisks mark the selected feature centroids by the first operator. Across all observers and tumours, a range of 0.95–6.7 pixels for euclidean distances between MRI and MSOT was calculated. This corresponds to a range from 71 μm to 503 μm in registration error.

Comparison of the anatomical similarity of the imaging data from the two modalities subjected to our co-registration framework was made in three K8484 tumour bearing mice. K8484 tumours were used for this purpose as they contain heterogeneous structural features visible in both MSOT and MRI. Upon visual inspection of images from three mice bearing this tumour type, it can be seen that the body shapes and tumour locations images demonstrate high anatomical similarity (Fig. 6). The pattern contrast in the MSOT Total Haemoglobin (THb) and MRI $T_{2}$ weighted ($T_{2}$w) images suggest these are necrotic areas of different characteristics. Haemorrhagic necrosis may produce high THb and low $T_{2}$w signal, while non-haemorrhagic necrosis is likely to show the opposite due to the lack of haemoglobin in the area. Considering the feature locations (defined as distinct features in MRI and MSOT images belonging to the same structure, denoted by red annotations in Fig. 6), we established that the relative distance between the centroids of features between modalities across three observers showed a close agreement (ranging from 0.95 to 6.7 pixels, 71 μm to 503 μm, when comparing euclidean distances of feature centroids as estimated registration error). The standard deviations of selected points and the resulting euclidean distances between MRI and MSOT across all three observers were 0.66, 0.41, and 2.03 pixels, for each tumour respectively. In Fig. 6, the red rectangle indicates the extent of the observed features in MRI/MSOT image pairs, whereas the red asterisk highlights the point selected by the first operator within the feature. This point was subsequently used for determining the relative distance between modalities.

**Fig. 7:**
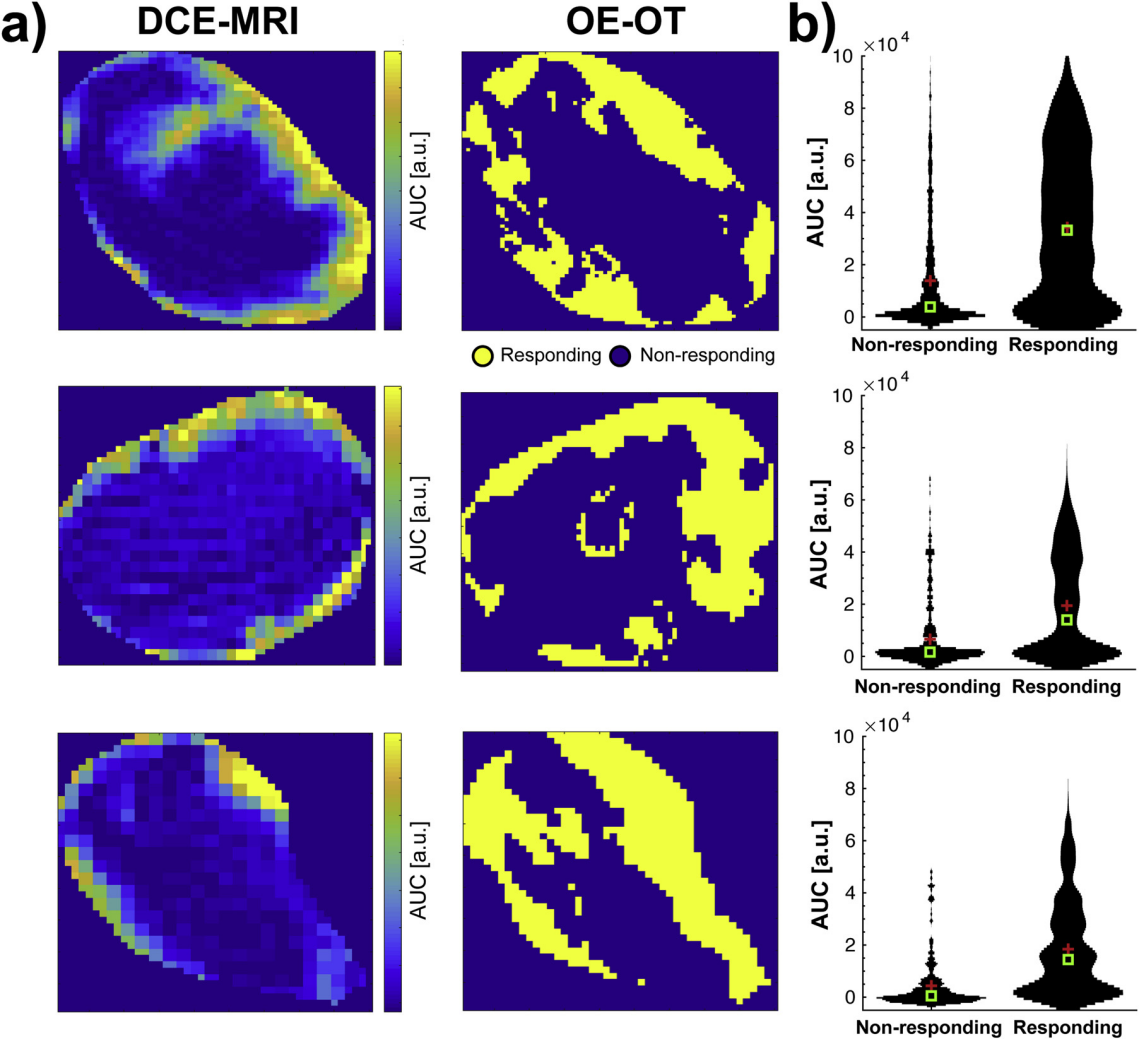
Measurements of vascular function show strong co-localisation in MRI and MSOT. (a) Maps of DCE-MRI area under the curve (AUC) enhancement 1 min after contrast injection (left) are in close spatial agreement with the maps of positive response to oxygen challenge in Oxygen-Enhanced Optoacoustic Tomography (right). (b) The DCE-MRI AUC is clearly higher in the areas positively responding in OE-OT. Mean and median are shown as red crosses and green squares, respectively. The width of the black lines indicates the distribution of values.

A comparison of functional imaging data was then made in the same K8484 tumours based on imaging data recorded using DCE-MRI and Oxygen Enhanced OT (OE-OT) protocols, which have been previously shown to relate to tumour perfusion and vascular function [6]. Visual comparison of DCE-MRI and OE-OT images (Fig. 7a) shows a similar distribution of perfused pixels in both modalities, with a greater number in the rim compared to the core of the tumour, as is commonly reported in subcutaneous xenografts. Quantitative comparison of DCE-MRI enhancement in regions of OE-OT response (Fig. 7b) shows a markedly stronger DCE-MRI enhancement in the areas showing positive response in the OE-OT, suggesting a functional relationship between these imaging biomarkers.

## 4 Discussion

Co-registration of images between modalities enables the combination of complementary information provided by different imaging methods. Due to deformation of the animal or patient between scans, correct alignment of images can pose a significant challenge and require both hardware and software-based optimisation approaches. In this work, we describe an integrated hardware and software framework for co-registration of mouse MSOT and MR imaging data. Without co-registration, these modalities produce very different images of the sample, due to different animal positioning and stress distribution.

On the hardware side, a novel silicone MRI animal holder was developed, which was designed to mimic the external stresses acting on the mouse body in the MSOT. Introducing the new holder alone already significantly improved the similarity in the shape of the entire mouse body contour as well as the individual tumour contour, contributing to more accurate co-registration. Importantly, the use of the holder did not increase animal preparation time or cause any side effects for animal welfare during imaging. Fabrication of the holder is simple and inexpensive, as soft two-component silicone is poured over a 3D printed mouse mold. The protocol offers a simple solution to improve MSOT/MR image co-registration.

A software tool for landmark-based image co-registration was then established to further improve the co-registration and enable per-pixel analysis of the combined multi-modal images. The transformation matrix for the MSOT images was calculated to maximise similarity between body and tumour outlines in both modalities as well as to minimise the distances between anatomical landmarks. The result of applying this software tool was a co-localisation error up to several 100 s of microns, comparable to the typical resolution of both modalities. The robustness of the landmark-based co-registration approach was verified by comparing the positioning of landmarks by several operators, yielding a close agreement. This framework also enabled per-pixel combination and comparison of the insight offered by MSOT and MRI in functional imaging. The relationship between tumour perfusion, provided by early DCE-MRI enhancement [9], and vascular function, given by the MSOT response to oxygen challenge [6], served as a proof of concept for further MSOT/MRI comparison. In the future, other algorithms introducing corrections for non-linear transformations such as compression and shear may be explored to further improve the internal co-registration. Due to increased number of degrees of freedom of the model, a careful validation of the results will have to be ensured to avoid over-fitting.

Despite the clear improvements in image co-registration achieved, there remain some limitations to our study. Firstly, the described two-step hardware and software framework is designed to aid with 2D co-registration, which assumes already the correct, manual choice of matching imaging slice between the modalities. The use of the silicone holder can help in this task, as the similar cross-sectional shape of the tumour in the MRI can help match it qualitatively to the geometry in the MSOT. Slice misalignment, although minimised with the use of the holder and an optimised slice registration protocol, can introduce additional error in the co-registration procedure.

A second limitation arises in the design of the silicone bed, which aimed to mimic the effects of the polyethylene film holder used in the MSOT, as well as the stresses due to water submersion during MSOT imaging. In order to support the weight of the animal, the silicone had to be stiffer than optimal, causing some discrepancy in MRI/MSOT mouse positioning. Further optimisation using silicones of different elastic properties could better match the distribution of forces and should be investigated in future experiments.

Finally, the silicone bed was created for a specific mouse size based on the typical usage in our experiments. If needed, additional silicone beds could be created to account for different mouse sizes, across strain and age for instance, and taking the individual tumour position into consideration. The optimal approach would utilize 3D modeling to create mouse-specific holders and require standardisation of the modelling, printing and casting workflow.

### 4.1 Conclusion

We have demonstrated the feasibility of a hardware- and software-based image registration framework for MRI and MSOT images. We use a novel silicone MRI holder, as well as a software tool to perform landmark-based co-registration of the images. Both steps led to a significant improvement in the registration of the tumour outlines and internal structure between the modalities. This simple, inexpensive approach can be readily implemented for multi-modal MSOT/MRI studies of mice, which will help to provide valuable insight into relative performance of these two modalities in revealing vascular architecture and function in cancer.
